# Transcriptome assembly, profiling and differential gene expression analysis of the halophyte *Suaeda fruticosa* provides insights into salt tolerance

**DOI:** 10.1186/s12864-015-1553-x

**Published:** 2015-05-06

**Authors:** Joann Diray-Arce, Mark Clement, Bilquees Gul, M Ajmal Khan, Brent L Nielsen

**Affiliations:** Department of Microbiology and Molecular Biology, Brigham Young University, Provo, UT 84602 USA; Department of Computer Science, Brigham Young University, Provo, UT 84602 USA; Institute of Sustainable Halophyte Utilization, University of Karachi, Karachi, Pakistan; College of Arts and Sciences, Qatar University, Doha, Qatar

**Keywords:** Halophytes, *Suaeda*, RNA-seq, Differential expression, Transcriptome profiling, De novo assembly, Transcriptome, Salt tolerance

## Abstract

**Background:**

Improvement of crop production is needed to feed the growing world population as the amount and quality of agricultural land decreases and soil salinity increases. This has stimulated research on salt tolerance in plants. Most crops tolerate a limited amount of salt to survive and produce biomass, while halophytes (salt-tolerant plants) have the ability to grow with saline water utilizing specific biochemical mechanisms. However, little is known about the genes involved in salt tolerance. We have characterized the transcriptome of *Suaeda fruticosa*, a halophyte that has the ability to sequester salts in its leaves. *Suaeda fruticosa* is an annual shrub in the family Chenopodiaceae found in coastal and inland regions of Pakistan and Mediterranean shores. This plant is an obligate halophyte that grows optimally from 200–400 mM NaCl and can grow at up to 1000 mM NaCl. High throughput sequencing technology was performed to provide understanding of genes involved in the salt tolerance mechanism. De novo assembly of the transcriptome and analysis has allowed identification of differentially expressed and unique genes present in this non-conventional crop.

**Results:**

Twelve sequencing libraries prepared from control (0 mM NaCl treated) and optimum (300 mM NaCl treated) plants were sequenced using Illumina Hiseq 2000 to investigate differential gene expression between shoots and roots of *Suaeda fruticosa*. The transcriptome was assembled de novo using Velvet and Oases k-45 and clustered using CDHIT-EST. There are 54,526 unigenes; among these 475 genes are downregulated and 44 are upregulated when samples from plants grown under optimal salt are compared with those grown without salt. BLAST analysis identified the differentially expressed genes, which were categorized in gene ontology terms and their pathways.

**Conclusions:**

This work has identified potential genes involved in salt tolerance in *Suaeda fruticosa*, and has provided an outline of tools to use for de novo transcriptome analysis. The assemblies that were used provide coverage of a considerable proportion of the transcriptome, which allows analysis of differential gene expression and identification of genes that may be involved in salt tolerance. The transcriptome may serve as a reference sequence for study of other succulent halophytes.

**Electronic supplementary material:**

The online version of this article (doi:10.1186/s12864-015-1553-x) contains supplementary material, which is available to authorized users.

## Background

Salinity affects about 400 million hectares of land worldwide due to excessive irrigation and continues to increase in parallel with the population. Salinity in soil and water has caused substantial economic losses, including an estimated $230 million for the Indus Basin in Pakistan and $2 billion for the Colorado River basin in the U.S. [1,2]. An estimated total of 200 million hectares of new cropland is needed to feed the rapidly expanding population but only 93 million hectares are available for expansion and farming of traditional crops [1]. Attempts have been made with conventional crops to breed salt tolerance; however, these crops can only tolerate limited amounts of salt in their systems. The potential of halophytes, the natural flora of saline habitats, has been under-examined until recently and their utilization may allow production of useful crops on salty soils.

*Suaeda fruticosa,* a succulent shrub in the family Chenopodiaceae, is an obligate halophyte that grows optimally at 300 mM NaCl and has the adaptation to reduce sodium buildup for long term survival [2]. This perennial halophyte has a strong ability to accumulate and sequester Na^+^ and Cl^−^ without the aid of salt glands, bladder or trichomes [3]. It is a good source of high quality edible oil [4], has potential for antiophthalmic, hypolipidaemic and hypoglycemic medicinal purposes [5], and has economic usage as forage for animals [6]. *S. fruticosa* also could help in bioremediation and reclamation of soils contaminated with toxic metals [7] and salinity [8]. Field studies showed that this plant can remove about 2646 kg of NaCl per hectare from the soil each year [9]. At optimum (300 mM NaCl) salt treatment of this species antioxidant enzymes trigger stress response through the activation of H_2_O_2_^−^ mediated Ca^2+^ uptake to maintain Na^+^ homeostasis at the cellular or tissue level [2]. Calcium ions, responsible for the overall signaling network of growth and development of the plant, are accumulated in the cell cytosol with the increase of Na^+^ [10]. At higher salinities, a significant reduction in growth is prevalent which might be due to the maximum threshold of the plant’s ability to adjust to specific ion toxicity and osmotic capability. Physiological data analysis has led to reports of ion accumulation, osmotic adjustments, maintenance of pressure potential and growth and production of glycinebetaine as part of a salt tolerance mechanism [11]. Previous studies of the impact of salinity on *S. fruticosa* have linked salt tolerance to its ability to uptake K^+^ in order to maintain a higher K^+^/Na^+^ ratio in the shoots. Higher sequestration of sodium and chloride in the shoot vacuoles together with the ability to synthesize osmoprotectants such as glycinebetaine has been suggested to maintain a favorable water potential gradient and protect cellular structures. Similar to *Suaeda fruticosa*, the majority of halophytes do not have glands or external bladders to modulate their tissue ion concentration therefore it has been seen to be a good model genus for the study of salt tolerance [12].

Next generation sequencing allows differential gene expression analysis of gene alleles and spliced transcripts, non-coding RNA and others, which will lead to identification of differentially expressed and/or unique genes. In this transcriptome paper, we report the identification of genes that are induced or repressed in plants grown under optimal salt conditions in comparison to low salt conditions. We generated a data set of transcript sequences from the roots and the shoots of *Suaeda fruticosa*. The genes were compared for differential expression under the indicated treatments using the assembled transcriptome, and common and tissue-specific patterns of transcriptomic responses were also analyzed. This first transcriptome study of *Suaeda fruticosa* expands our knowledge on global gene expression data for salt-accumulating halophytes that do not have external bladders.

## Results and discussion

### De novo transcriptome assembly and assessments of expressed sequenced tags

#### Experimental design

To prepare for the transcriptome assembly and analysis, total RNA was extracted from shoots and roots of *Suaeda fruticosa*. These include biological triplicates of cDNA libraries for *S. fruticosa* roots from plants grown without salt (R000), roots with 300 mM optimal salt (R300), shoots with no salt (S000) and shoots with 300 mM optimal salt (S300). Total mRNA was purified using oligo dT and transcribed into cDNA libraries using TruSeq RNA Sample Prep Kit for Illumina 100 bp paired-end sequencing.

#### Sequencing method and quality assessment of the reads

A total of 335.3 million reads of 100 bp were generated by Illumina Hi-seq platform (Sequence Read Archive Accession Number: SRX973396). The reads were filtered using Trimmomatic to remove adapters, FASTX toolkit and Sickle program to remove low quality reads and discard reads based upon the threshold of length. A total of 84.58% of the reads were trimmed and filtered totaling to 283,587,292 reads.

**Figure 1:**
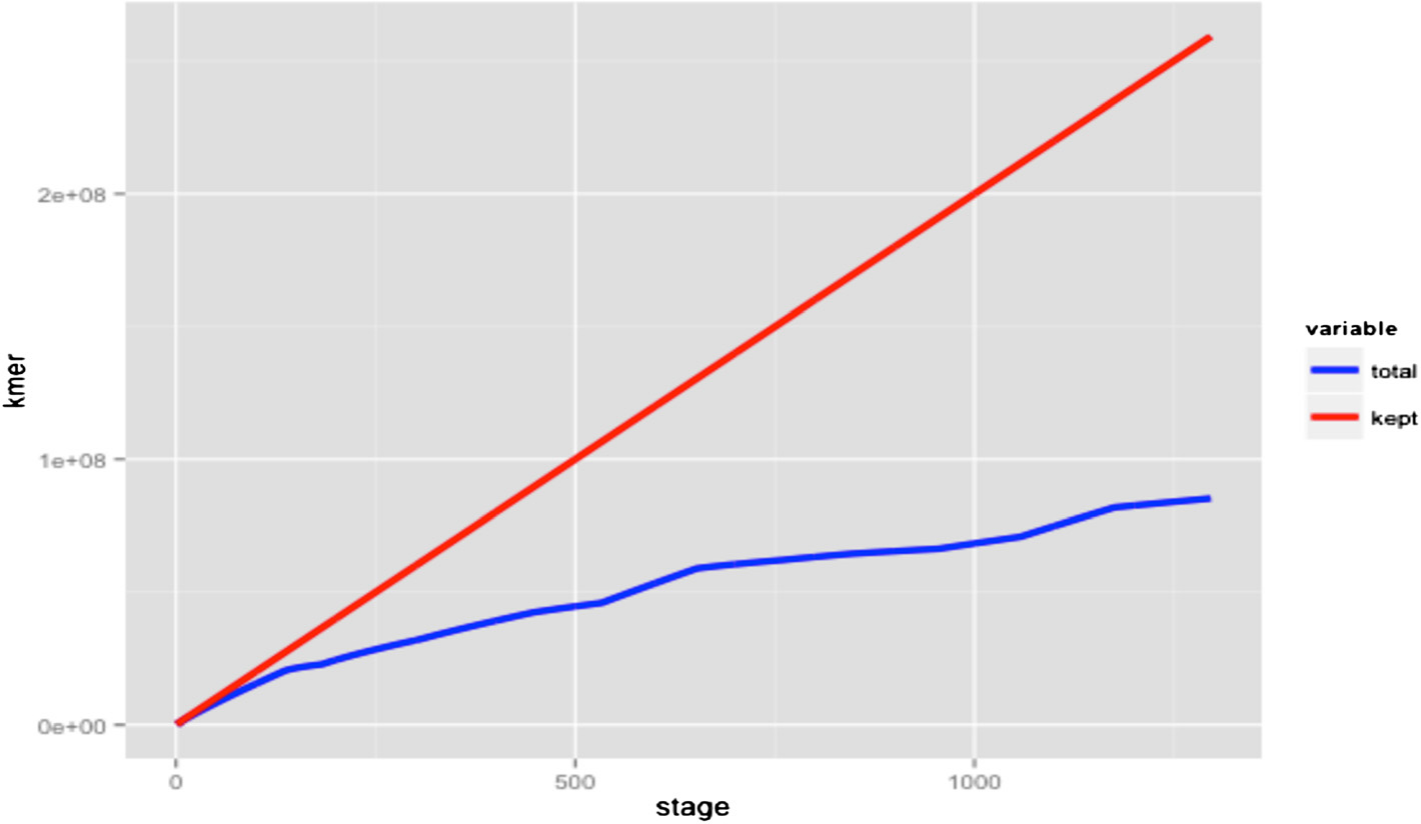
Plot of total read pairs versus kept read pairs after digital normalization algorithms. The true *k-mer* counts are kept using digital normalization to reduce computational memory and correct redundancy.

To normalize and assemble RNA-seq reads for *de novo* assembly, digital normalization was used for 283.6 million reads. *K*-mer hash of 21 with coverage of 30X was built from a set of reads to correct redundancy issues, variations in sequences, and potential errors among the reads. Since some reads with sequencing errors may escape quality score-based filtering steps, the reads with potential errors are flagged for removal from the dataset to improve the de novo assembly. Sequencing errors can affect the assembly algorithms so it is best to eliminate the reads that have non-uniform *k*-mer coverage. Reads that have non-uniform k-mer coverage create a problem with the assembly therefore it is necessary to normalize the reads to a certain threshold. This threshold represents the approximate minimum for de novo assembly to work optimally and efficiently. Digital normalization [13] was applied to the total of 283,587,292 paired end reads with *k*-mer size of 21 and k-mer coverage cutoff of 30X. The retained reads were normalized to 99,577,045 (Table 1) to remove overabundant reads, reduce noise of the sequenced sample and decrease the overall percentage of errors (Figure 1). The effect of digital normalization is to retain nearly all real *k*-mers while discarding the majority of erroneous and redundant k-mers. This step allows reducing the reads and obtaining a transcriptome assembly much faster than and superior to the assembly based on the full data set without affecting the quality of the assembly. Because the genes in the transcriptome have different levels of expression, k-mer distribution will not show any peak at any k value.

**Table 1:** **Statistics of reads**

**Reads preparation**	**Libraries**	**Number of reads**	**Total reads**
Raw reads	R000	95,248,764	335,271,656
	S000	75,414,804	
	R300	84,162,958	
	S300	80,445,130	
FastX toolkit and Trimmomatic	R000	68,444,064	292,898,120
	S000	68,872,348	
	R300	79,313,812	
	S300	76,267,896	
Sickle Trimmed	All		283,587,292
Digital Normalization	All		99,577,045

The summaries of the pre-assembly methods are indicated. R000 represents roots in 0 mM NaCl treatment, S000 are shoots in 0 mM NaCl treatment, R300 are roots in 300 mM NaCl treatment and S300 are shoots in 300 mM NaCl treatment.

To assemble the *Suaeda fruticosa* transcriptome we utilized a genome-independent reconstruction approach. The strategy involved building a de Bruijn graph made of overlapping subsequences or *k*-mers using Velvet [14]. The overlapping bases allow building a graph of all the sequences that then traverse a path guided by read and paired-end coverage [15]. The path through the graph is reported as transcripts. To assemble the contigs into scaffolds, we used a de Bruijn graph software, Oases [16]. *K*-mer sizes from 35 to 99 were chosen to generate the assemblies. We assessed the quality of the assemblies based on the total number of transcripts, open reading frames predicted using Transdecoder and highest mapping percentage of the reads using GSNAP. The number of sequences, N50 values, mean length of the sequences and total base pair length for the contigs, scaffolds and unigenes were also determined (Table 1). Among the individual *k*-mer values, transcript numbers associated with different *k-*mer values vary from 1 to 450,588. *K*-mer length is related inversely to the number of transcripts generated. The highest N50 (computed by sorting the contigs from largest to smallest and then determining the minimum set whose sizes total 50% of the assembly) generated for the transcripts is 1,755 generated by a *k*-mer length of 59. Predicted open reading frames range from 1 to 108,112 bp with the highest number of ORFs being generated with a *k-*mer of 41. The highest N50 for open reading frames belongs to an assembly with a *k*-mer of 65 with 1,272 bp. Mapping coverage for the assemblies range from 30.39% to 72.91%. The highest percentage of reads mapping back to the assembly belongs to assemblies with k-mers 41 and 45 with 72.91% and 72.61% mapped. Assembly *k*-45 contains a higher percentage of proper pairs aligned with 59.56% and higher N50 compared to Assembly *k*-41, therefore it was chosen to be the assembly for the succeeding steps.

Assembly k-45 contained 296,776 contigs from Velvet with a N50 length of 1548 bp and mean size of 928 bp. We selected contigs that were greater than 200 bp in length. The contigs were assembled into scaffolds using Oases and yielded 273,824 contigs with an N50 length of 1669 bp and mean size of 1012 bp. The shortest scaffold is 152 bp and the longest one is 14,046 bp. Using CDHIT-EST, scaffold sequences were assembled into clusters and Transdecoder was used to predict open reading frames. The sequences were further screened for adapter and vector contamination using NCBI VecScreen and the UniVec database. Those sequences that are longer than 200 bp were kept. Using this pipeline, we obtained 54,526 high-quality unigenes with an N50 of 957 bp. The size range of the unigenes is between 200 to 6639 bp. There are 12,914 unigenes comprising 23.7% of the total that have lengths of more than 1000 bp. The mean size of the unigenes is 763 bp (Table 2).

**Table 2:** **Statistics of sequence assembly**

	**Contigs**	**Scaffolds**	**Unigenes**
Number of sequences	296,766	273,824	54,526
N50 (bp)	1,548	1,669	957
Mean length (bp)	928	1,012	764
Total length (bp)	275,319,083	277,056,733	41,651,347

The table shows the summary of de novo sequence assembly after using Velvet for contig assembly, Oases for Scaffolds then CDHIT-EST and Transdecoder for the unigenes determination.

### Functional annotation, gene ontology assignments and analysis

**Figure 2:**
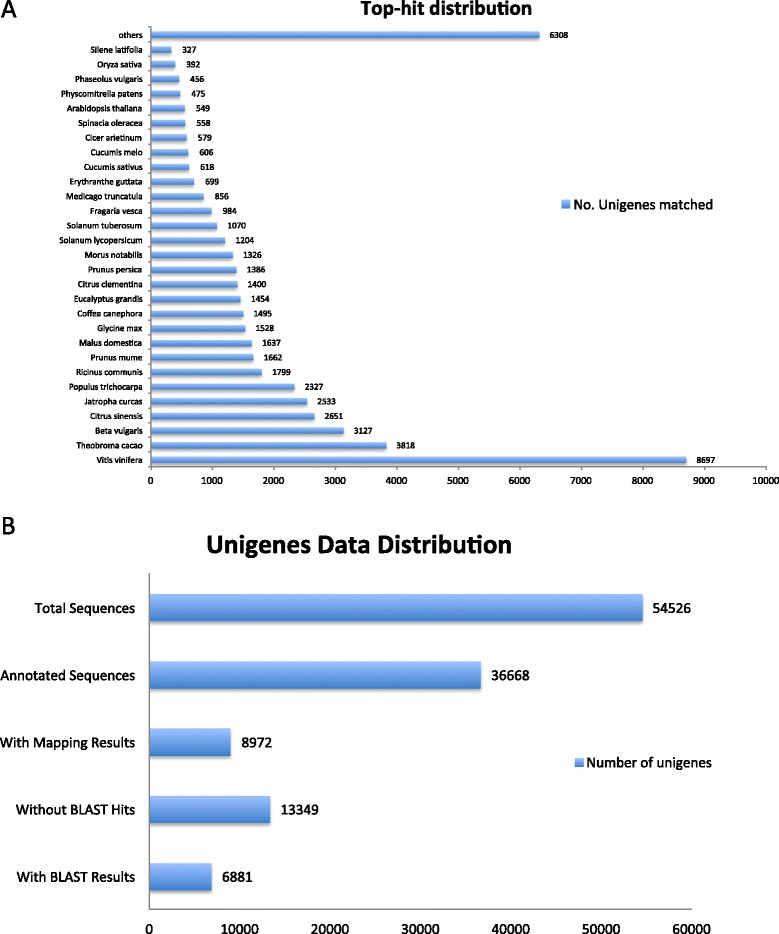
Top hit distribution of matched unigenes among different species generated from BLASTX. **(A)**. Data distribution summary from BLAST2GO shows blast hits, mapping results and annotated sequences **(B)**.

The unigenes assembled were used as query for annotation using BlastX searches based on sequence homologies to the databases of the National Center for Biotechnology Information (NCBI) non-redundant (nr) protein database, RefSeq, SwissProt UniProt and the Kyoto Encyclopedia of Genes and Genomes (KEGG) using BLAST2GO. The summary of top hit distribution similar to *Suaeda fruticosa* unigenes is illustrated in Figure 2A. The species distribution with the lowest e-value matching the best sequence alignment result showed that the *S. fruticosa* transcriptome sequences have 8697 unigenes (16%) matching to *Vitis vinifera* (grapes), 3818 unigenes (7%) matching to *Theobroma cacao* (cacao tree) and 3127 unigenes (5.7%) similar to *Beta vulgaris* (beet). The closest halophyte species matching with *Suaeda fruticosa* is *Populus trichocarpa* (poplar tree) with 2327 unigenes (4.3% matching). For poplar only the initial analysis of the draft genome has been completed; additional mapping and sequencing is ongoing. Some of the halophytes mentioned in this paper do not have full annotation of genes submitted to NCBI database and some only contain partial transcriptome information. Figure 2B summarizes the data distribution summary from the sequences from the assembled transcriptome.

Gene names and GO terms were assigned to the transcripts based on homologies with an E-value threshold of 10^-10^. The data distribution summary for these sequences is shown in Figure 2B. Annotated sequences utilize assigned functional terms to query sequences from GO terms based on the gene ontology vocabulary. Mapped sequences are those with retrieved GO terms associated with the hits obtained after a BLAST search. The search produced 36,668 annotated sequences among 54,526 total transcripts, comprising 67.25% of total sequences. There are 8972 sequences comprising 16.45% of the total transcripts that did not surpass the annotation threshold and 6881 sequences or 12.6% had hits in the databases but lack functional information. A large proportion has no significant sequence alignment or hits in any of the databases, comprising 13,349 sequences or 24.5% of total transcripts which suggests that they may contain novel sequences or a high number of *Suaeda fruticosa* specific transcripts or transcript portions such as orphan untranslated regions.

**Figure 3:**
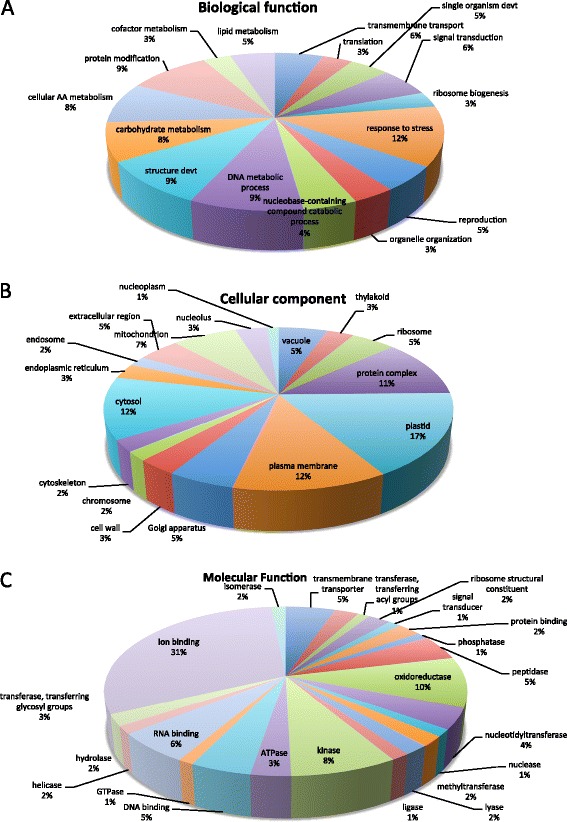
Gene Ontology Summary of total assembled ESTs using BLAST2GO. Distribution of Gene Ontology Annotation of *Suaeda fruticosa* transcriptome. The results are summarized as follows: **(A)** Biological Process, **(B)**. Cellular component **(C)** Molecular Function.

Gene ontology encompasses a dynamic library for gene and protein roles in cells. This includes three main categories: Biological process, referring to the biological objective of the genes or gene products; Molecular function, defined by the biochemical activity of the genes or gene products; and Cellular components, referring to the place in the cell where the gene product is active [17]. Figure 3 illustrates the gene ontology annotation of the total assembled unigenes from the de novo assembled transcriptome of *Suaeda fruticosa* using BLAST2GO.

In the biological process category, genes related to stress make up 1229 of the total unigenes annotated (Figure 3A). The other dominant subcategories were protein modification (933 unigenes), structural development (930 unigenes) and DNA metabolic process (923 unigenes). The following subcategories include unigenes involved in carbohydrate metabolism (798 unigenes), nucleobase-containing compound catabolic process (443 unigenes), organelle organization (348 unigenes), reproduction (513 unigenes), ribosome biogenesis (319 unigenes), signal transduction (594 unigenes), single organism development (457 unigenes), translation (346 unigenes), transmembrane transport (556 unigenes), lipid metabolism (513 unigenes), cofactor metabolism (314 unigenes), and cellular amino acid metabolism (850 unigenes). Figure 3B illustrates the cellular component category, which has a dominant subcategory of plastid (1374 unigenes), plasma membrane (1003 unigenes) and protein complex (941 unigenes). The molecular function category was comprised of protein coding genes involved in ion binding (3061 unigenes), oxidoreductase (961 unigenes), and those responsible for redox reactions of the cell and kinases (809 unigenes) (Figure 3C). These gene ontology annotations represent a profile for gene expression of *Suaeda fruticosa* suggesting that this species has diverse protein coding genes comprising its structural, regulatory, metabolic and stress response mechanisms.

### Differential expression analysis

**Figure 4:**
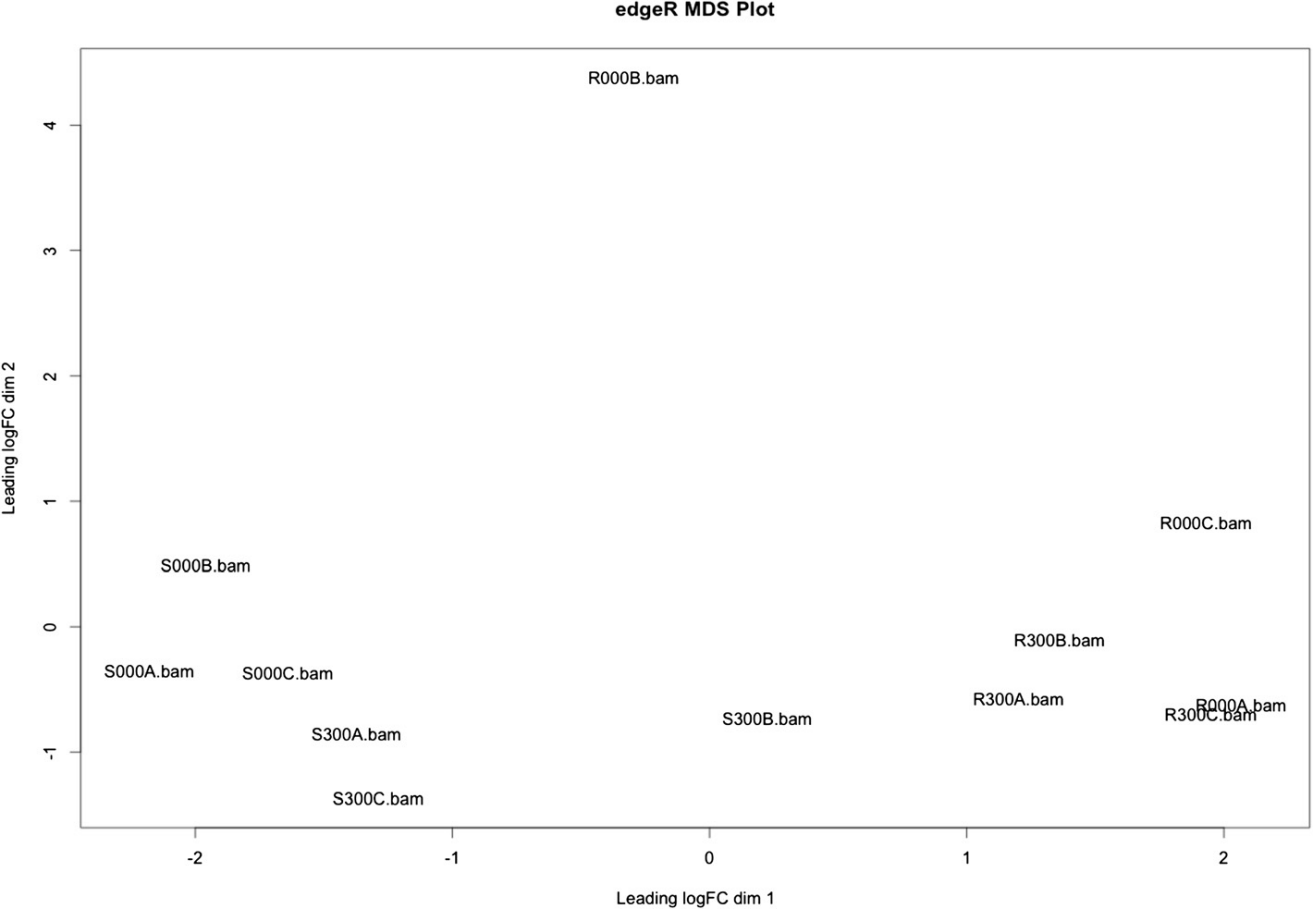
Multidimensional Scaling Plot for the sequencing libraries. Multidimensional Scaling Plot (MDS) is designed to indicate sample relationship similarity. Shoots and roots of 0 mM and 300 mM NaCl with their biological replicates are analyzed. (Key: S000A- shoots 0 mM replicate A, S000B- shoots 0 mM replicate B, S000C- shoots 0 mM replicate C, S300A- shoots 300 mM replicate A, S300B- shoots 300 mM replicate B, S300C- shoots 300 mM replicate C, R000A- roots 0 mM replicate A, R000B- roots 0 mM replicate B, R000C- roots 0 mM replicate C, R300A- roots 300 mM replicate A, R300B- roots 300 mM replicate B, R300C- roots 300 mM replicate C, .bam (bam files)).

To acquire counts data for differential expression analysis, samples of different treatments (0 mM and 300 mM NaCl treatments) were mapped to the newly generated reference transcriptome using GSNAP (Genomic Short-Read Nucleotide Alignment Program) which utilizes computational methods to detect variants and splicing isoforms in short reads through merging and filtering position lists from a genomic index. It also detects short and long-distance splicing including interchromosomal splicing using probability models or a database of known splice sites [18]. Conversion of bam files into count data was performed using BamBam [19] to summarize the number of reads mapped to each annotated feature. Differential expression calls were made using the EdgeR package. Normalization is applied to the treatments and tissue types to provide accurate differential expression rather than individual quantification. The EdgeR package adjusted the analysis taking into account sequencing depths represented by library sizes. Variations between biological replicates were clustered closely using a multidimensional scaling (MDS) plot similar to that shown in Figure 4 to check for variations among replicates and samples.

**Figure 5:**
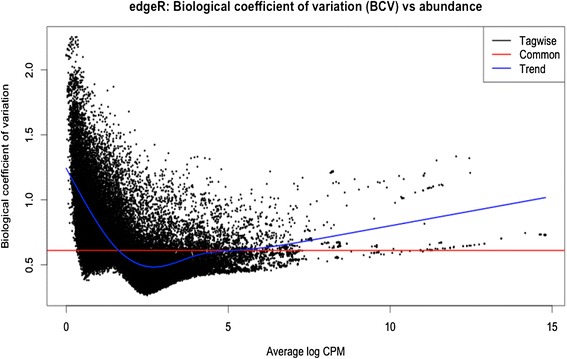
Biological coefficient of variation plot. Genewise dispersion plot for twelve libraries is indicated. Estimation of genewise BCV allows observation of changes for genes that are consistent between biological replicates and giving less priority to those with inconsistent results. Generalized linear model is used to determine the evidence of significant difference of counts for a transcript or exon across conditions. The BH method is used in this dataset to control false discovery rate.

The replicates of treatments and their tissue types from transcriptome analysis were used to produce a multidimensional scaling plot (Figure 4), which allows us to see a spatial configuration of how similar or dissimilar the different treatments and biological replicates of *S. fruticosa* shoot and root samples are. The relationship of shoot treatments is more closely clustered together in comparison to root treatments. The tight clustering of the shoot data points means there are fewer variations among biological replicates in comparison to the root treatments. Root samples, however, have greater variations among the treatments and their biological replicates. This indicates that root tissues show less consistency with expression of genes among treatments. Common dispersions were then estimated on the distributions of reads across genes. Each gene gets an assignment of a unique dispersion estimate, which is to be compared to a common dispersion. The biological coefficients of variation versus the abundance were plotted (Figure 5). This specifies relative abundance of each gene variation between RNA samples and also measurement error estimated by the sequencing technology. From this sample, it shows a common dispersion of 0.37 and BCV of 61.09%. This means that common variation shows overall variability across the genome for this dataset and the common variation square root indicates high coefficient of biological variation. The genewise dispersions show a decrease at low average log counts per million. It indicates that at low expression level of genes or transcripts, the variability of gene abundance is high.

**Figure 6:**
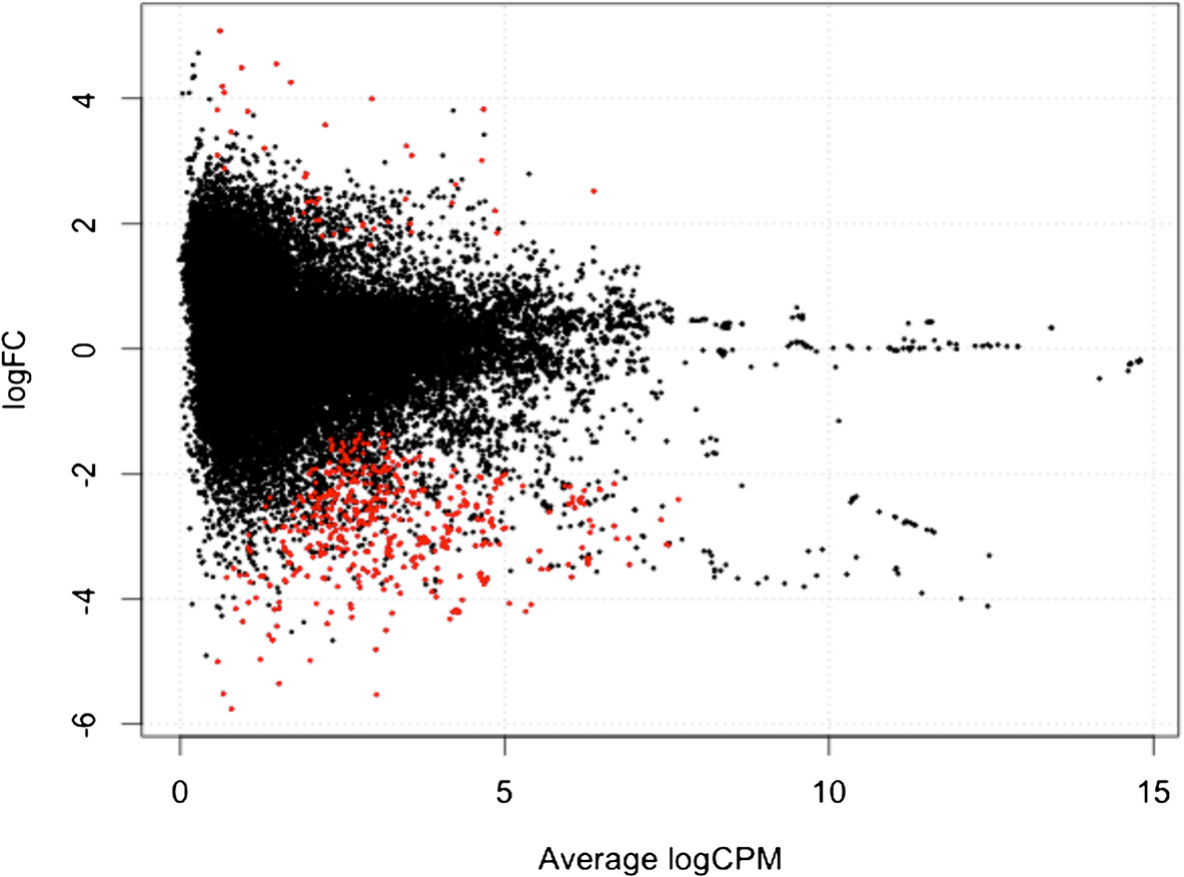
Differential expression genes plot. Plot of LogFCs against average count size, highlighting the differentially expressed genes in red. From the samples and the replicates, there are 475 genes identified to be downregulated and 44 genes that are upregulated with p-value of <0.05 and FDR rate of <0.05.

The analyses were concentrated on genes that are significantly different in expression levels in the optimum salt transcriptome as compared to the low condition transcriptome. Genes whose adjusted p-values were less than 0.05 using the BH method were considered differentially expressed [20,21]. The BH method known also as FDR (false discovery rate) by Benjamini, Hochberg, and Yekutieli enables the user to control the false discovery rate, the expected proportion of false discoveries amongst the rejected hypotheses. The false discovery rate is a less stringent condition than the family-wise error rate, so these methods are more powerful than the others. RNA-seq gene expression for *Suaeda fruticosa* is visualized as an MA plot (log ratio versus abundance plot) in Figure 6. The red dots highlight transcripts that are differentially expressed among biological replicates and treatments. There are 475 genes that are downregulated and 44 genes are upregulated with a p-value <0.05 and false discovery rate <0.05. The results are consistent with the physiological data of *Suaeda fruticosa* [11] where at 0 mM NaCl treatment, more genes are downregulated in comparison to optimal growth of 300 mM NaCl.

### Gene annotation and identification of differentially expressed genes

The differentially expressed genes were annotated using Blast2GO software against NCBI non-redundant protein database with a cut-off E-value of 10^-10^. Enrichment analysis was performed for the biological functions of the identified DEGs. Among 519 differentially expressed unigenes, 44 of them are upregulated upon salt treatment and 475 are downregulated. These genes were identified from BLAST nr, SwissProt and UniProt databases and assigned with Gene Ontology terms in biological process, molecular function and cellular component categories.

The top hit species distribution of these differentially expressed genes included grapes (*Vitis vinifera*) with 48 unigenes, orange (*Citrus sinensis*) with 35 unigenes, and *Theobroma cacao* with 29 genes. The closest halophyte is *Populus trichocarpa* with 13 unigenes and *Mesembryanthemum crystallinum* with 9 unigenes. Draft genome projects for both *P. trichocarpa* and *M. crystallinum* are currently ongoing while other halophytes only have partial transcriptome information available in the NCBI database.

**Figure 7:**
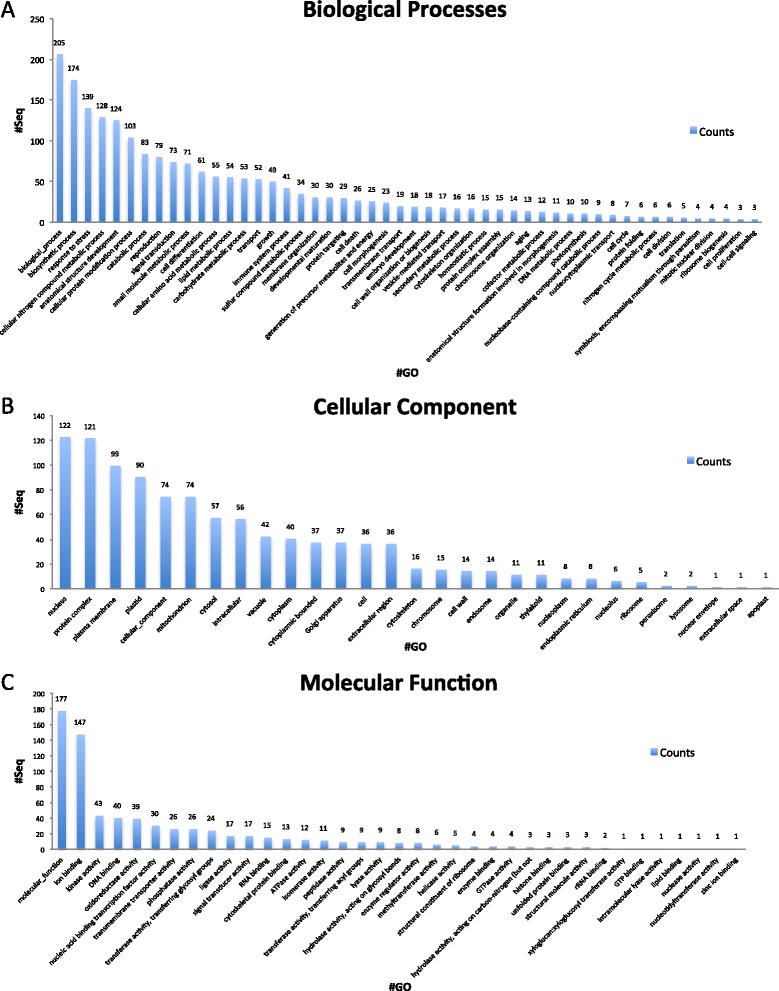
Summary of differentially expressed ESTs using BLAST2GO. Differentially expressed transcripts were classified into 3 main GO annotations: Biological Processes **(A)**, Cellular Component **(B)** and Molecular Functions **(C)**. There are 25 GO terms for biological processes, 37 GO for molecular function and 15 GO for cellular component. A majority assigns the GO from biological process as stress response genes, genes responsible for oxidation-reduction and structure development. A few transcripts reflect oxidoreductase and kinase activity for molecular function. A majority of the transcripts is distributed to the nucleus and plasma membrane.

From 519 differentially expressed genes, 391 unigenes have significant BLAST hits (75%) and the remaining 25% do not have any significant sequence alignments, which suggests that they might be genes that are novel or have not been reported in any other plant databases. There were 371 annotated sequences (71.5%), and 282 have InterProScan matches from the European Bioinformatics Institute (EBI) that can be mapped to GO terms and annotations while 162 of them have been assigned to gene ontology IDs. The summary in Figure 7 shows how many genes are assigned to at least one GO term and grouped into three main GO categories: biological processes (A), cellular component (B), and molecular function (C). Direct GO terms from Blast2GO were performed by counting annotated sequences in each term and suggesting the top terms. Among these sequences, 177 total unigenes are identified in the molecular function category of GO annotation. The top hits included genes functioning in ion binding (147 unigenes), kinase activity (43 unigenes) and DNA binding (40 unigenes). In the cellular component category, the top hits are genes found to be active in the nucleus (122 unigenes), protein complex component (121 unigenes) and plasma membrane (90 unigenes). For the biological process category, 205 unigenes have been assigned with GO terms and GO IDs. There are 174 unigenes that are important in biosynthetic process, 139 unigenes responding to stress and 124 unigenes involved in cellular nitrogen compound metabolic process. The differentially expressed genes were assigned to KEGG to identify pathways that these genes might be involved in related to salt tolerance. Among the annotated differentially expressed unigenes, the top hit included 6 sequences that are involved in both nitrogen and histidine metabolism. Others function in lysine degradation, glycerolipid metabolism and linoleic acid metabolism. Other pathways are illustrated in Additional file 1.

### Relative gene expression validation using qRTPCR analysis of RNA from 0 mM and 300 mM samples

**Figure 8:**
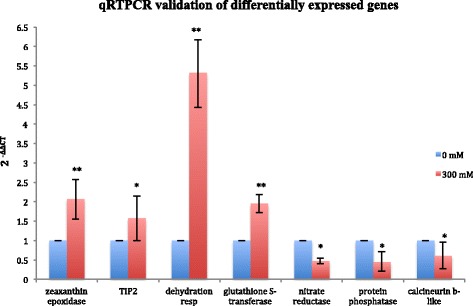
qRTPCR validation of the transcriptome data. Each panel shows the qRTPCR results for seven test genes. The annotated putative genes are listed on the x-axis and the mean fold change represented by the 2^-ΔΔCT^ method relative to 0 mM treated samples are shown on the y axis. Error bars depict the standard error of the mean for 3 biological replicates. Significant differences (p < 0.05) are denoted with an asterisk and highly significant differences with p-value of <0.005 are represented with double asterisks.

To validate the results from the transcriptome analysis, we selected seven differentially expressed genes with putative functions related to salt tolerance. Specific primers were designed and optimized using PCR for the selected DE genes and for alpha tubulin as the endogenous control (Additional file 2). We amplified a cDNA library from six samples of 0 mM treated plants and six 300 mM treated samples. Analysis of transcript levels by qRTPCR showed that expression for all seven gene targets selected correspond with the differential expression patterns determined from the transcriptome analysis. Four targets (zeaxanthin epoxidase, aquaporin TIP2, dehydration responsive protein and glutathione S-transferase) show upregulation of mRNA expression while the other three targets (nitrate reductase, putative protein phosphatase and calcineurin B-like (CBL) 4–1) show downregulation upon 300 mM NaCl treatment compared to the absence of salt treatment (Figure 8).

### Putative salt tolerance-related genes

BLAST analysis data identified a large number of differentially expressed genes and we have grouped them in the following categories: 1. Genes responsible for enzymes, transcription factors, hormones, photosynthetic genes, detoxifiers and osmolytes for general metabolism, 2. Genes functioning as transporters for water and ion uptake, 3. Genes involved in regulation such as kinases and phosphatases, and 4. Genes that function to protect the cells against abiotic stress such as late embryogenesis abundant protein, heat shock proteins, osmoprotectants such as dehydrins and osmotins. The number of transcripts reported to be differentially regulated or expressed depends on the conditions being compared. In this study, we are comparing transcript expression between 0 and 300 mM NaCl treatment and their biological replicates. Upregulated genes are those with significant increased expression when treated with salt (300 mM). Those downregulated are annotated sequences with decreased expression with salt treatment. A summary of these sequences, their definitions and putative functions, and references from other halophytes or plants is shown in Table 3.

**Table 3:** **Summary of selected differentially expressed genes in** ***Suaeda fruticosa***

**Differentially expressed Protein--‐coding genes** ^**1**^	**Putative function or role in salt tolerance**	**Expression upon 300 mM salt treatment (upregulated or downregulated)**	**Plants with orthologous genes**	**References**
**General metabolism genes**				
Probable WRKY transcription factor 72	Sequence specific DNA binding transcription factor; Activators of ABA signaling Repressors of aleurone cells	Upregulated	*Festuca rubra ssp litoralis*	[22,25,69]
			*Glycine soja*	
			*Glycine max*	
			*Oryza sativa*	
			*Porteresia coarctata*	
WRKY transcription factor 6--like	Influence senescence and pathogen defense--‐associated PR1 promoter activity; mediates arsenate/phosphate transporter gene expression	Downregulated	*Arabidopsis thaliana*	[22,23,25,26]
			*Festuca rubra ssp litoralis*	
			*Glycine soja*	
			*Glycine max*	
			*Porteresia coarctata*	
WRKY DNA binding protein isoform 2	Transcription factors involved in various regulations; crucial to salinity tolerance	Downregulated	*Festuca rubra ssp litoralis*	[22-25,69]
			*Glycine soja*	
			*Glycine max*	
			*Porteresia coarctata*	
Gibberellin 2--beta--‐dioxygenase 2 family (GA2OX2)	Gibberellin catabolic process; response to jasmonic acid and red light	Downregulated	*Arabidopsis thaliana*	[28,70,71]
40S ribosomal protein S4 (RPS4)	Disease resistance; SRP--‐dependent cotranslational protein	Upregulated	*Arabidopsis thaliana*	[72,73]
			*Mesembryanthemum crystallinum*	
60S acidic ribosomal protein P2	Elongation step of protein synthesis	Upregulated	*Zea mays*	[30,74]
60S ribosomal protein L18–2--‐like	Plastidic and nuclear protein synthesis	Upregulated	*Populus euphratica*	[31,34]
			*Suaeda maritima*	
Pre--mRNA processing protein 40C	Coactivator involved in the regulated transcription of nearly all RNA polymerase II--‐dependent genes	Downregulated	*Arabidopsis thaliana*	[30]
DNA--binding protein escarola--‐like	Late flowering and leaf development Leaf senescence	Upregulated	*Arabidopsis thaliana*	[30]
MADS--box transcription factor AGL24	Transcription activator that mediates floral transition in response to vernalizationl promotes inflorescence fate in apical meristem	Downregulated	*Arabidopsis thaliana*	[23,30]
			*Porteresia coarctata*	
DNA binding protein with zinc finger isoform1	Binds DNA; structural regulation	Downregulated	*Glycine max*	[30,69,75]
			*Malus zumi*	
			*Arabidopsis thaliana*	
F--box protein AT1g78280--‐like transferase	Regulation of transcription; defense response by callose deposition	Downregulated	*Arabidopsis thaliana*	[30,34,76]
			*Eutrema salsugineum*	
			*Suaeda maritima*	
Glutathione--S--‐transferase tau 1	Glutathione metabolism; and production; promoted a higher level of salt tolerance	Upregulated	*Arabidopsis thaliana*	[2,31-33,35,36,69,77,78]
			*Glycine max*	
			*Glycine soja*	
			*Nicotiana tabacum*	
			*Populus euphratica*	
			*Reaumuria trigyna*	
			*Salicornia europaea*	
			*Suaeda maritima*	
			*Suaeda fruticosa*	
			*Suaeda salsa*	
Germin--like protein	Salt--‐stress regulation marker	Upregulated	*Arabidopsis thaliana*	[37,38]
			*Hordeum vulgare*	
Flowering promoting factor 1--like protein 3	Regulates flowering time	Upregulated	*Arabidopsis thaliana*	[30]
Auxin--induced protein 5NG4--‐like	Transport of molecules functioning downstream of the auxin response; Root formation	Upregulated	*Arabidopsis thaliana*	[30]
Pathogenesis--related protein	Defense response; Response to water deprivation;	Upregulated	*Arabidopsis thaliana*	[30]
Chitinase	Enhance biotic and abiotic stress tolerance; reduce chitin in the cell wall contributing to salt sensitivity	Upregulated	*Glycine max*	[40,69]
			*Glycine soja*	
			*Nicotiana tabacum*	
Peroxisomal ascorbate peroxidase (APX)	Response to oxidative stress; Regeneration of NAD+; induced by high temperature	Upregulated	*Nicotiana tabacum*	[30,41]
			*Zea mays*	
Plant cadmium resistance 2--like	Reduces cadmium accumulation	Upregulated	*Atriplex halimus*	[42-44]
Chaperone protein DNAJ–16 like	Protein folding; protein partitioning into organelles; signal transduction; directly interacts with HSP70; induced by heat shock and prevents apoptosis	Downregulated	*Arabidopsis thaliana*	[34,45,46,79,80]
			*Atriplex nummularia*	
			*Suaeda maritima*	
			*Spartina maritima*	
			*Spartina alterniflora*	
Ethylene--responsive transcription factor rap2--‐7 like isoform	Transcriptional activator; binds to GCC box pathogenesis related promoter; Involved in the regulation of gene expression by stress factors; negatively regulates transition to flowering time	Downregulated	*Arabidopsis thaliana*	[30,34]
			*Suaeda maritima*	
Senescence--associated protein	Induced by abscisic acid; regulated during natural and artificially induced leaf senescence	Downregulated	*Arabidopsis thaliana*	[72,81]
			*Mesembryanthemum crystallinum*	
Stem specific protein TSJT1--like	Stem--‐specific (active at lower levels in other organs)	Downregulated	*Nicotiana tabacum*	[30]
Protein F3H11–7	Positive regulation of transcription; leaf morphogenesis	Downregulated	*Arabidopsis thaliana*	[30]
Cell wall protein AWA1--like	Cell wall organization and biosynthesis	Downregulated	*Theobroma cacao*	[30]
Callose synthase 7	Callose synthesis at forming cell plate during cytokinesis; transitory component of the cell plate in dividing cells	Downregulated	*Arabidopsis thaliana*	[30]
Calcineurin B--like protein (CBL) 4--‐1	SOS like--‐gene; Acts as a calcium sensor involved in regulatory pathway for the control of Na + and K+ homeostasis and salt tolerance; Activates in synergy with CIPK24/SOS2 to activate Na+/H+ antiporter SOS1	Downregulated	*Arabidopsis thaliana*	[47,82,83]
			*Eutrema salsugineum*	
Calmodulin binding isoform 1	Regulates transcriptional activity in response to calcium signals; activates the expression of the V--‐PPase proton pump in pollen	Downregulated	*Arabidopsis thaliana*	[47,48,69,84]
			*Glycine max*	
			*Glycine soja*	
			*Leymus chinensis*	
Photosystem II protein z (PsbZ)	Controls photosystem II cores with the light-­‐harvesting antenna	Upregulated	*Arabidopsis thaliana*	[30,72,75]
			*Malus zumi*	
			*Mesembryanthemum crystallinum*	
Photosystem D2 protein chloroplastic (psbD)	One of the two reaction center proteins of photosystem II; needed for assembly of a stable PSII complex	Upregulated	*Arabidopsis thaliana*	[30]
Photosystem II CP43 chlorophyll apoprotein (psbC)	Component of the core antenna complex of photosystem II; binds chlorophyll and helps catalyze the primary light--‐induced photochemical processes of photosystem II	Upregulated	*Arabidopsis thaliana*	[30]
Hypothetical Chloroplast RF19 (ycf1)	Unknown; may have a function which is probably not related to photosynthesis.	Upregulated	*Arabidopsis thaliana*	[30]
Zeaxanthin epoxidase, Chloroplastic--like isofom *X*2	Precursor to abscisic acid; involved in response to salt and heavy metal tolerance; required for resistance to osmotic and drought stresses, ABA--‐dependent stomatal closure, seed development and dormancy, modulation of defense gene expression; disease resistance	Upregulated	*Arabidopsis thaliana*	30,80]
			*Spartina maritima*	
			*Spartina alterniflora*	
Sufe--like chloroplastic like	Cysteine desulfurization in chloroplast and mitochondria; iron--‐sulfur cluster biosynthesis	Upregulated	*Arabidopsis thaliana*	[30]
High Light--induced chloroplastic protein	Possible role in chlorophyll and/or carotenoid binding	Downregulated	*Arabidopsis thaliana*	[30]
CRS2--associated factor chloroplastic like	Required for the splicing of group IIB introns in chloroplast; mRNA processing; intron specificity	Downregulated	*Arabidopsis thaliana*	[30]
			*Zea mays*	
Thioredoxin--like protein z chloroplastic like	Redox regulation in the apoplast; cell division; cell differentiation; pollen germination; stress responses	Downregulated	*Reaumuria trigyna*	[32,80]
			*Spartina alterniflora*	
			*Spartina maritima*	
Triose phosphate chloroplastic like isoform *X*2	Exports photoassimilates from chloroplast; transports inorganic phosphate, 3--‐phosphoglycerate and triose phosphate	Downregulated	*Arabidopsis thaliana*	[30]
Probable chlorophyll b reductase chloroplastic--like	Chlorophyll B degradation	Downregulated	*Arabidopsis thaliana*	[30]
Phosphate chloroplastic like	Hypothetical protein	Downregulated	*Arabidopsis thaliana*	[30]
**Ion transporters**				
Aquaporin tonoplast intrinsic protein 1	Water channel; facilitates the transport of water across cell membrane; osmoregulation; hydrogen peroxide transmembrane transport	Upregulated	*Arabidopsis thaliana*	[30,31,52,53,69,75,85]
			*Glycine max*	
			*Glycine soja*	
			*Malus zumi*	
			*Oryza sativa*	
			*Populus euphratica*	
			*Schrenkiella parvula*	
High affinity nitrate transporter 3.1 like	High--‐affinity nitrate transport; repressor of lateral root initiation; nitrate assimilation; response to wounding	Upregulated	*Arabidopsis thaliana*	[30]
			*Oryza sativa*	
Nitrate transporter 1.5	Transmembrane nitrate transporter; involved in xylem transport of nitrate from root to shoot; induced in response to nitrate	Upregulated	*Arabidopsis thaliana*	[30]
Aluminum--activated malate transporter 10	Malate transporter critical for aluminum tolerance; response to aluminum ion	Upregulated	*Arabidopsis thaliana*	[30]
Flavonol 4’--sulfotransferase, putative	Auxin transport; catalyze the sulfate conjugation	Upregulated	*Flaveria chlorifolia*	[30]
Bidirectional sugar transporter SWEET3	Mediates low affinity uptake and efflux of sugar across the plasma membrane	Upregulated	*Arabidopsis thaliana*	[30]
Glucosyltransferase	Catalyzes the glycosylation of flavonoids from UDP glucose	Upregulated	*Arabidopsis thaliana*	[30,31]
			*Populus euphratica*	
			*Populus pruinosa*	
Seed storage/lipid transfer protein	Bifunctional inhibitor/lipid transfer protein/seed storage 2S albumin superfamily	Upregulated	*Arabidopsis thaliana*	[30,31,86]
			*Populus euphratica*	
			*Thellungiella halophila*	
ATPase subunit 1 (chloroplast)	Maintainance of the pH of endomembrane compartments	Upregulated	*Arabidopsis thaliana*	[30]
			*Leymus chinensis*	
			*Thellungiella halophila*	
Sodium HKT1--like	Plant salt tolerance; response to salt and osmotic stress; involves in Na + recirculation; potassium ion transmembrane transporter	Downregulated	*Arabidopsis thaliana*	[30,32,33,53,86]
			*Populus trichocarpa*	
			*Reaumuria trigyna*	
			*Salicornia europaea*	
			*Schrenkiella parvula*	
			*Thellungiella halophila*	
			*Thellungiella salsuginea*	
Sodium pyruvate chloroplastic cotransporter	Pyruvate transport across chloroplast envelope	Downregulated	*Arabidopsis thaliana*	[30]
Magnesium transporter NIPA2	Magnesium ion transmembrane transport; can transport other divalent cations	Downregulated	*Arabidopsis thaliana*	[30]
Vacuolar Iron transporter family	Regulation of iron distribution; cellular response to ethylene stimulus; cellular response to nitric oxide; iron ion homeostasis; ion transport	Downregulated	*Arabidopsis thaliana*	[30]
Vacuolar proton ATPase A1--like	Essential component of the vacuolar proton pump (V--‐ATPase); cell expansion; ATP hydrolysis	Downregulated	*Arabidopsis thaliana*	[30,84]
			*Leymus chinensis*	
ATPase ASNA1 homolog	Required for the post--‐translational delivery of tail anchored proteins to the endoplasmic reticulum; binds the transmembrane domain of tail--‐anchored proteins in the cytosol	Downregulated	*Arabidopsis thaliana*	[30]
Glutamyl--tRNA amidotransferase subunit chloroplastic mitochondrial--‐like	Allows the formation of correctly charged Gln--‐tRNA; ATP binding; glutaminyl--‐tRNAGln biosynthesis; mitochondrial translation	Downregulated	*Arabidopsis thaliana*	[30]
Tonoplast dicarboxylate transporter--like protein	Malate transmembrane transport; critical for pH homeostasis; indirectly involved in the uptake of malate and fumarate to the vacuole	Downregulated	*Arabidopsis thaliana*	[30]
Probable Galacturonosyltransferase 12-like	Involved in pectin assembly and/or distribution; cell wall organization	Downregulated	*Arabidopsis thaliana*	[30]
**Regulatory molecules**				
Cysteine rich receptor like protein kinase	ATP binding; defense responses; disease resistance	Upregulated	*Arabidopsis thaliana*	[30]
Phosphatase 2C family protein	Stress responses; metal ion binding; protein dephosphorylation; Serine/threonine phosphatase activity	Downregulated	*Arabidopsis thaliana*	[30,31,58,69,80,87]
			*Ceriops tagal*	
			*Glycine max*	
			*Glycine soja*	
			*Populus trichocarpa*	
			*Spartina maritima*	
			*Spartina alterniflora*	
			*Thellungiella salsuginea*	
Phosphatase 2C 76 isoform 1	Metal ion binding; Binds 2 magnesium or manganese ions	Downregulated	*Arabidopsis thaliana*	[30]
CDPK related kinase 1	Signal transduction pathways that involve calcium as second messenger; ATP binding; Ca2 + binding; protein autophosphorylation	Downregulated	*Arabidopsis thaliana*	[30,34,75,76]
			*Eutrema salsugineum*	
			*Malus zumi*	
			*Suaeda maritima*	
PERK1 receptor protein kinase	Protein autophosphorylation; response to wounding; ATP binding	Downregulated	*Arabidopsis thaliana*	[30]
Casein kinase I--2--‐like protein	ATP binding; protein serine/threonine kinase activity	Downregulated	*Arabidopsis thaliana*	[30]
Serine--threonine protein kinase (histidine transporter) HT1	Control stomatal movement; shows a reduced response to ABA or light	Downregulated	*Arabidopsis thaliana*	[30,57]
			*Oryza sativa*	
Phosphotidylinositol 4--kinase gamma 4	Phosphatidylinositol phosphorylation; Response to salt stress; Protein autophosphorylation	Downregulated	*Arabidopsis thaliana*	[30]
Serine threonine protein phosphatase pp1--like	Binds 2 manganese ions per subunit; protein dephosphorylation; serine/threonine phosphatase activity	Downregulated	*Arabidopsis thaliana*	[30]
Serine threonine--protein phosphatase PP2A catalytic subunit	Metal ion binding; serine/threonine phosphatase activity	Downregulated	*Arabidopsis thaliana*	[30]
**Late embryogenesis abundant proteins**				
Dehydration--responsive RD22--‐like	Induced by salt stress; stress response	Upregulated	*Malus zumi*	[88]
			*Populus euphratica*	
			*Populus pruinosa*	
HSP20--like chaperones superfamily protein	Associated with stress and other abiotic factors	Downregulated	*Glycine max*	[89,90]
			*Oryza sativa*	

^1^Identified and annotated using BLAST nr database using BLAST2GO.

#### General metabolism genes

Genes that are involved in transcription, translation and post-translational modification have been seen to play roles in salt tolerance processes. WRKY transcription factors are important regulators for signaling mechanisms that modulate various plant processes. It has been found to interact with protein partners, MAP kinases, calmodulin, histone deacetylases, resistance proteins for autoregulation and transcriptional reprogramming [22]. It has also been suggested to be crucial for salinity tolerance [23]. From the differential expression analysis of the transcriptome, we have found WRKY transcription factor 72 to be significantly upregulated while WRKY transcription factor 6-like and WRKY DNA-binding protein isoform 2 are downregulated. The salt tolerant grass *Festuca rubra ssp litoralis* was found to have a differentially regulated WRKY-type transcription factor in response to salinity [24]. Transient expression studies have also found OsWRKY72 and OsWRKY77 to be activators of ABA signaling but repressors of gibberelic acid signaling in aleurone cells [25]. Moreover, in Arabidopsis, AtWRKY6 negatively autoregulates its own promoter to influence senescence and pathogen defense-associated PR1 promoter activity. This targets SIRK, a gene encoding a receptor-like protein kinase that is strongly induced during leaf senescence. The activation of SIRK is dependent on WRKY6 function [26]. These studies suggest that WRKY72 trancription factor is upregulated to respond to ABA signaling, important for stress tolerance while downregulating protein-coding genes involved in senescence for protection and defense. Gibberelic acid (GA) genes, which regulate many aspects of growth and development of plants, are involved in the synthesis of gibberellin hormone. In Arabidopsis, reduction of bioactive GA is shown via an increase in gibberellin 2-oxidase 7 (GA2ox7). This leads to accumulation of DELLA proteins, which are transcriptional regulators that repress GA-responsive growth and development, inhibiting plant growth [27]. Downregulation of GA2ox2 is observed at 300 mM salt treatment in *Suaeda fruticosa*. This suggests that the decrease deactivates bioactive GA [28]. GA genes were regulated at 200 mM NaCl in *S. europaea* similar to homologues of gibberellin 3-oxidase and gibberellin 20-oxidase in *P. trichocarpa*. Two DELLA domain GRAS family transcription factors were downregulated in plants treated with 200 mM salt [29].

Both 40S ribosomal protein S4 and 60S ribosomal protein L18-2-like that are upregulated in *S. fruticosa* are part of a group of SRP-dependent co-translational proteins targeting to membranes responsible for translation and protein binding [30]. Ribosomal protein 40S and 60S and RNA binding family protein are also highly upregulated in this transcriptome study. Similar studies were performed in *Poplar euphratica,* which found that ribosomal 60S rRNA, important for plastidic and nuclear protein synthesis, is increased in response to salinity [31]. 60S acidic ribosomal protein P2, known to play an important role in the elongation step of protein synthesis and other RNA-binding family proteins are upregulated in 300 mM NaCl treated *Suaeda fruticosa*. However, the gene encoding pre-mRNA processing protein 40C undergoes downregulation in salt treated plants. This protein has been found to be a coactivator involved in regulated transcription of RNA polymerase II-dependent genes important in transcription and other regulatory mechanisms [30]. Some DNA binding proteins also show concerted regulation upon salt treatment. DNA-binding escarola-like protein responsible for late flowering and leaf development and F-box kelch repeat protein AT1g80440-like are upregulated while MADS-box transcription factor AGL24, an early target of transcriptional repression at floral transitional stage, DNA-binding protein with zinc finger isoform1 and F-box protein AT1g78280-like transferase involved in regulation of transcription are downregulated.

An increase in reactive oxygen species causing damage to cellular components is evident when salinity increases. Genes that are responsible in regulating redox reactions are usually involved in protecting the cell environment during these stresses [32]. Upregulation of glutathione-S-transferase tau 1 (GST) and glutathione transferase were seen in the differential expression analysis of *S. fruticosa*. Similarly, glutathione S-transferases were greatly increased upon salt treatment in roots of the halophyte *Salicornia europaea* [33], *Suaeda maritima* and *Reaumuria trigyna* [32,34]. The *Suaeda salsa* GST gene was introduced into *Arabidopsis* and improved salt tolerance after overexpression in transgenic plants. Glutathione content increased in salt-stressed *Arabidopsis* and promoted a higher level of salt tolerance [35]. The level of glutathione is increased at 0 mM and 900 mM treatment and decreased at the optimal condition of 300 mM NaCl in *S. fruticosa* [2].

A similar trend of higher salt tolerance is seen in tobacco seedlings upon overexpression of GST and these genes have been found to be responsible for increased protection against toxins [36]. Some proteins important for seed production and growth show differential expression in *S. fruticosa*. Germin-like protein, found to be an important plant marker for salt stress regulation and suggested to undergo change when salt-tolerant plants are subjected to salt stress has been found to be significantly upregulated upon salt treatment [37,38]. An ortholog of flowering promoting factor 1-like protein 3, which promotes flowering in *Arabidopsis*, and auxin-induced protein 5NG4-like gene involved in transport of molecules functioning downstream of the auxin response and responsible for root formation are also upregulated [39]. Some genes encoding proteins involved in protection such as pathogenesis-related protein, chitinase, peroxisomal ascorbate peroxidase (APX), and plant cadmium resistance 2-like are also increased. Plant chitinase plays an important role in plant defense and enhances resistance and tolerance to heat, salt and drought [30]. Overexpression of chitinases in transgenic tobacco has been shown to enhance biotic and abiotic stress tolerance [40]. In tobacco cells, APX functions in the regeneration of NAD+ and is usually induced by high temperature stress and functions against toxic reactive oxygen species [41]. In the halophyte *Atriplex halimus L*., chloride salinity reduces cadmium accumulation as salinity resistance is found to be closely associated with the gene loci responsible for cadmium extraction [42-44]. Proteins containing chaperone domains and DNAJ-16 like chaperon protein are also decreased upon salt treatment. The DNAJ protein family is included in the group of heat shock proteins functioning as molecular chaperones, and is associated with HSP70 and involved in resisting environmental stresses in *Suaeda maritima* [34]. Specifically DNA-J16 in *Arabidopsis* is encoded by the gene known as Altered Response to Gravity 1 (ARG1), and mediates gravity signal transduction and hypocotyl gravitropism [45,46]. Other genes that are downregulated include ethylene-responsive transcription factor rap2–7 like isoform, senescence-associated protein, stem specific protein TSJT1-like, root hair protein F3H11–7, cell wall protein AWA1-like isoform X1 for cell wall organization, and callose synthase 7, a major component of pollen tubes and pollen cell walls. Molecular mechanisms of cellular calcium changes have been seen with the downregulation of calcineurin B-like protein (CBL) and calmodulin binding isoform 1 upon salt treatment suggesting their potential role as regulators of salt and drought responses [47]. Calmodulin mediates auxin signaling and responds to stresses in *Arabidopsis* [48]. CBL interacts with CIPK serine-threonine protein kinases and mediates activation of AKT1 in response to low potassium conditions and stomatal movement [49].

Various photosynthetic genes have been found to be differentially upregulated upon salt treatment in *S. fruticosa*. These include genes encoding photosystem II protein z, d2 protein, cp43 chlorophyll apoprotein, chloroplast RF19, zeaxanthin epoxidase, chloroplastic like isoform *X*2 and sufE-like chloroplastic protein. Significant induction has also been found in the halophyte *Salicornia europaea* in which photosynthetic genes, PSI and PSII pigment binding proteins, b6f complex and ATPase synthase CF1 are upregulated in salt treated plants [33]. Some genes encoding light-induced chloroplastic protein, CRS2-associated factor, thioredoxin-like protein chloroplastic like, triose phosphate chloroplastic-like isoform *X*2, probable chlorophyll b reductase chloroplastic-like and phosphate chloroplastic-like are downregulated in *S. fruticosa*. While some of these proteins have no definite functions determined yet, chlorophyll b reductase has been found to play a role in maturation and storability of seeds in *Arabidopsis. Arabidopsis* plants lacking chlorophyll b show a stay-green phenotype in leaves [50]. This suggests that as chlorophyll b reductase decreases in plants, they tend to prevent chlorophyll degradation.

#### Ion transporters (transporters and aquaporins)

Homeostasis of the cellular environment involves the maintenance of cellular uptake to control ionic balance. Since a large influx of extracellular Na + occurs in halophytes, plants require high amounts of K+ (100–200 mM) to lower the amount of Na + and maintain osmosis [51]. Aquaporin tonoplast intrinsic proteins showed upregulation in salt treated *S. fruticosa*. Aquaporins are membrane proteins that facilitate uptake of soil water and mediate regulation of root hydraulic conductivity. They are also involved in compartmentalization of water and are found in halophytes to play a role in maintaining osmosis and turgor of plant cells [52]. The halophyte *Schrenkiella parvulla* contains high numbers of aquaporins for tolerance to boron toxicity [53]. In *Poplar* species, some aquaporins are decreased to prevent water loss during salt stress [31]. Some other transporters that are upregulated upon salt treatment include high-affinity nitrate transporter 3.1-like and nitrate transporter 1.5 important for nitrate uptake, aluminum-activated malate transporter 10 for increased aluminum tolerance, flavonol 4-sulfotransferase for auxin transport, bidirectional sugar transporters and glucosyltransferase for glucose and other sugar transport, seed storage/lipid transfer protein responsible for metabolism and transport, and ATPase subunit 1. Other halophytes such as *Schrenkiella parvula* and *Thellungiella* showed upregulation of genes encoding for ATPases that are necessary for large influx of ions [53,54].

Studies have shown vacuolar Na+/H+ antiporter to be important for salt tolerance through Na + sequestration [55]. However, in *Suaeda fruticosa*, sodium transporter HKT1-like is shown to be downregulated. In *Arabidopsis*, HKT1 knockouts accumulate the highest concentration of Na + in the shoots suggesting a role in maintenance of Na + concentration [56]. Some other ion transporters are also downregulated such as sodium pyruvate chloroplastic co-transporter, magnesium transporter NIPA2, vacuolar iron transporter, vacuolar proton ATPase A1-like and ASNA1 (arsenic pump driving ATPase). Glutamyl-tRNA amidotransferase involved in carbon-nitrogen ligase activity, tonoplast dicarboxylate transporter-like protein for malate transmembrane transport and regulation of intracellular pH and galacturonosyltransferase 12-like for glycan and pectin biosynthesis are also decreased with salt treatment.

#### Regulatory molecules (kinases and phosphatases)

Differentially regulated molecules such as kinases and phosphatase are involved in regulation of proteins involved in osmolyte synthesis and detoxification by oxidants. They are suggested to play a role in ionic and osmotic homeostasis and modulate ion transport for salt tolerance [57]. Cysteine-rich receptor like protein kinase, phosphatase 2C family protein including phosphatase 2C 15-like isoform X1 and purple acid phosphatase 27-like are upregulated at 300 mM NaCl treatment. Protein phosphatase 2C (PP2C) regulates signal transduction pathways. In *Thellungiella*, A-type PP2C phosphatases are generally upregulated in response to abscisic acid [58]. Moreover, there are other kinases that are downregulated in this study such as CDPK-related kinase 1, PERK1 kinases, casein kinase I2-like protein, and serine-threonine protein kinase HT1 and phosphoinositide 4-kinase gamma 4. Serine threonine protein kinase HT1 is important for regulation of stomatal movement in response to carbon dioxide [59] while CDPK-kinase 1 has been shown to play an important role in mediating signal transduction of growth and development [30]. In rice, OsCDPK1 negatively regulates the expression of enzymes for gibberellic acid biosynthesis. This also transduces post-germination of Ca^2+^ signal from sugar starvation and gibberellic acid to prevent drought stress injury [60]. Some phosphatases are also downregulated such as serine-threonine protein phosphatase pp1-like, phosphatase 2C 76 isoform 1 and PP2A catalytic subunit. In *Arabidopsis*, transcription factor MYB20 negatively regulates 2C serine-threonine protein phosphatases to enhance salt tolerance [61].

#### LEA genes

Late embryogenesis abundant (LEA) proteins comprise a group of proteins that have crucial roles in cellular dehydration tolerance. They have been associated with tolerance to dehydration caused by freezing, salinity or drying. During stress conditions such as salinity, plant hormone abscisic acid (ABA) is produced to develop tolerance against drought. Some genes are induced to trigger the production of ABA [62].

Overexpression of LEA proteins can improve stress tolerance of transgenic plants [62]. In this transcriptome study, salt treatment causes upregulation of dehydration-responsive RD22-like protein. RD22 expression in *Arabidopsis* is mediated by abscisic acid (ABA). This is also induced by salt stress and dehydration [63] and is expressed during early and middle stages of seed development. Housekeeping gene HSP20 chaperone superfamily is found to be downregulated upon salt treatment. HSP20 family has been associated with the most stress-general expression pattern including salt stress in *Arabidopsis* [64].

## Conclusions

This study provides an overview of the genes present in a non-model plant species and identifies the genes associated with salt tolerance. The assembled transcriptome was used for differential expression studies and gene annotations. We have identified 519 genes that are differentially expressed based on p-value and adjusted false discovery rate of less than 0.05. The same pattern of differential expression for seven of these genes was confirmed by qRT-PCR analysis, which each showed similar levels of up- or down-regulation (Figure 8).

The annotation of genes using next generation sequencing is more readily available through the advancement of technology. Analysis of predicted genes allows assumptions to be made on the complexity of genetic mechanisms for this plant. RNA sequencing generates an enormous amount of data in terms of identifying the transcripts, however the challenges remain with the analysis. One of the major problems is the development of expression metrics that will allow comparisons of different expression levels and also provide identification of differentially expressed genes. We have utilized a combination of approaches to conduct this analysis for the *Suaeda fruticosa* transcriptome. The reference transcriptome assembly was not previously available and this species does not have any close relative plant that can serve as a basis for the expression analysis.

This study reports comprehensive information about the transcriptome of the succulent halophyte *S. fruticosa*. This will provide a basis for further study of the mechanism of salt tolerance, discovery of novel genes involved and comparison of expression profiles with no salt and optimal salt concentration. The de novo transcriptome generated in this study provides a useful source of reference sequence for succulent halophytes.

## Methods

### Plant materials and RNA isolation

Seeds of *Suaeda fruticosa* obtained from the Institute of Sustainable Halophyte Utilization, University of Karachi, Pakistan were planted and grown at Brigham Young University, Provo, Utah, U.S.A. according to protocol [2]. Plant samples of 100 mg of frozen plant tissue from roots and shoots of low (0 mM NaCl) and optimal (300 mM NaCl) salt conditions were ground in liquid nitrogen to a fine powder. Total RNA was extracted from these tissues using a Trizol-based method or QIAGEN RNeasy Mini kit. The RNA was analyzed for quality and concentration using the Agilent Technologies 2100 Bioanalyzer. High quality total RNA samples should give two distinct peaks and yield an RNA Integrity Number (RIN) value greater than 8.

### Illumina sequencing platform

The Illumina RNA-Seq library preparation protocol includes poly-A RNA isolation, RNA fragmentation, reverse transcription to cDNA using random primers, adapter ligation, size-selection from a gel and PCR enrichment [65]. The batch of libraries was sequenced at the BYU sequencing center and by Otogenetics (Norcross, GA) using Illumina Hi-seq 2000 sequencer. This includes cDNA libraries of Suaeda 0 mM NaCl-treated shoot and roots in triplicates and cDNA libraries of Suaeda 300 mM NaCl-treated shoot and roots in triplicates. The paired-end library was developed according to the protocol of the Paired-End sample Preparation kit (Illumina, USA).

### Bioinformatics analysis

#### Quality trimming and digital normalization

The adapters of raw RNA sequence data were trimmed using Trimmomatic v.0.27. FastX toolkit and Sickle paired-end trimmers were used to determine low quality reads towards the 3’ and 5’ ends of the reads. The software for digital normalization is available electronically through http://ged.msu.edu/papers/2012-diginorm/. A python script was used to interleave the paired-end reads file https://github.com/ged-lab/khmer/tree/2012-paper-diginorm/sandbox. Khmer software package available at https://github.com/ged-lab/khmer/tree/2012-paper-diginorm was used to perform three-pass normalization steps. Loading sequences needed for khmer software works with screed packages through https://github.com/ged-lab/screed/ (khmer and screed are ©2010 Michigan State University, and are free software available for distribution, modification, or redistribution under the BSD license). The details of quality trimming and digital normalization are available in Additional file 3.

#### De novo assembly and gene ontology

The high quality concatenated reads of shoots and roots (Sequence Read Archive Accession Number: SRX973396) were assembled using Velvet v. 1.2.10 and Oases v. 0.2.08 with optimized determined *k*-mer size of 45 with an average insert length of 300 bp and minimum contig length of 200. Further screening of sequences for vector contaminations and adapters were performed using NCBI Univec and VecScreen. Only assembled transcripts longer than 200 bp were kept. De novo assembly scripts are available in Additional file 3. We ran the clustering methods using CDHIT-EST v.4.5.4–2011–03–07 on the assembly. All Illumina assembled unigenes were searched against nr database in NCBI, Swiss-Prot, UniProt, and Kyoto Encyclopedia of Genes and Genome (KEGG) with the BLASTX algorithm. The E-value cut-off was set to 10^-10^. Genes were identified according to best hits against known sequences. Prediction of gene ontology (GO) terms, sequences functions, metabolic pathways in KEGG databases were performed.

#### Sequence analysis

The assemblies were transferred into Transdecoder, an open-reading frame predictor software under the Trinity package, which reports candidate coding regions within the transcripts. For each assembly, the number of transcripts, N50 values and the total length of the assemblies are identified. The analysis of the efficiency of assemblies is performed using GMAP and GSNAP v. 2013–11–27. GMAP maps and aligns cDNA sequences originally used for genomic mapping then GSNAP aligns single-end or paired-end reads. It can detect short and long distance splicing using probabilistic models or database of known splice sites.

#### Differential expression analysis

To determine the DEGs (differentially expressed genes) between different treatments of shoots and roots of *Suaeda fruticosa*, gene expression level analysis was performed using the EdgeR package from R [66]. Calculated gene expression can be directly used for comparing the differences in gene counts between treatments and tissue types. Generalized Linear Models were used for data analysis to take account of different salt conditions and tissue types of biological replicates. This determines the evidence of significant difference of counts for a transcript or exon across experimental conditions. The estimation for biological variation is measured. DEGs were identified and subject to further annotation using BLAST2Go.

#### Validation of differentially expressed genes through qRTPCR

Several putative annotated genes were selected for validation of differential expression using qRTPCR. These include aquaporin TIP2, protein phosphatase, calcineurin b-like protein (CBL) 4–1, zeaxanthin epoxidase, dehydration responsive protein, glutathione S-transferase and nitrate reductase. We selected alpha tubulin as an endogenous control. The primers for these genes were designed from the *Suaeda fruticosa* transcriptome sequences and optimized for PCR (Additional file 2).

For each qRTPCR reaction, 1 ug of RNA of 0 mM and 300 mM NaCl treated samples were reverse transcribed into cDNA using oligo (dT) primers, and the cDNA libraries produced were used for qRTPCR using the method of Haddad et al. [67]. To assess validation for each gene, qRTPCR data were analyzed based on ΔΔCT and 2^-ΔΔCT^ method [68]. The ΔCT value of each gene was calculated by subtracting the CT value of the endogenous control from the CT value of the target gene. Each gene’s mean ΔΔCT value.

2^-ΔΔCT^ and standard error of the mean were calculated using the data analysis package in Microsoft Excel. Data were plotted as mean fold change (2^-ΔΔCT^). Significant differences (p < 0.05) were determined using a one-tailed two sample *t*-test assuming equal variances for comparison of the fold change values between groups using GraphPad software.

### Availability of supporting data

Raw Illumina sequences are available at NCBI Sequence Read Archive under *Suaeda fruticosa* accession SRX973396 (SRA run accessions: SRR1946790, SRR1946833, SRR1946834, SRR1947647, SRR1947648, SRR1947649, SRR1947655, SRR1947656, SRR1947661, SRR1947683, SRR1947686, SRR1947688). Transcriptome sequence information is deposited in the Transcriptome Shotgun Assembly Sequence Database: BioProject ID: PRJNA279962 and PRJNA279890. The following supplementary information files are publicly available at LabArchives (DOI: http://dx.doi.org/10.6070/H4Z03650). Additional file 1 shows the exported name list of assembled sequences, their annotated names, gene ontology terms, associated enzymes and KEGG maps generated by BLAST2GO. Additional file 2 shows the summary of the number of unigenes assigned to different pathways. Additional file 3 lists the codes used for Bioinformatics analysis. Additional file 4 lists the primers used for qRTPCR on selected genes.

## Additional files

Additional file 1:
**List of assembled sequences, their annotated names, gene ontology terms, associated enzymes and KEGG maps.**
Additional file 2:
**Summary of the number of unigenes assigned to different pathways.**
Additional file 3:
**Codes for bioinformatics analysis.**
Additional file 4:
**List of primers for qRTPCR confirmation.**

## Abbreviations

R000: Roots treated without salt
R300: Roots treated with 300mM NaCl
S000: Shoots treated without salt
S300: Shoots treated with 300mM salt
ORF: Open reading frame
nr: Non-redundant database
GO: Gene ontology
KEGG: Kyoto Encyclopedia of genes and genomes
BLAST: Basic local alignment search tool
GSNAP: Genomic short read nucleotide alignment program
MDS: Multidimensional scaling plot
BCV: Biological coefficient of variation
FDR: False discovery rate
BH: Benjamini, Hochberg
GA: Gibberelic acid
GST: Glutathione S-transferase tau 1
APX: Peroxisomal ascorbate peroxidase
PP2C: Protein phosphate 2C
LEA: Late embryogenesis abundant proteins
ABA: Abscisic acid
DEG: Differentially expressed genes
