# Supplementary material for: Transcriptome assembly, profiling and differential gene expression analysis of the halophyte Suaeda fruticosa provides insights into salt tolerance
Source: BMC Genomics. 2015 May 6;16(1):353. doi: 10.1186/s12864-015-1553-x (PMC4422317; doi:10.1186/s12864-015-1553-x)
Supplement: Additional file 2: — Summary of the number of unigenes assigned to different pathways. [file 12864_2015_1553_MOESM2_ESM.docx]

Supplementary File 4

Primers for qRTPCR

Atubulin FWD CACGCGCTGTATTCGTAGAT

Atubulin REV TGACCACGAGCGAAGTTATTAG

Calcineurin b-like  FWD GACCTAAGGGAAACTGGCTTTA

Calcineurin b-like  REV ACAATGGCCTCCACTATATCATC

Dehydration-response FWD GGCGTCATCAAGCAACAAAG

Dehydration-response REV CGTACCGTCTTCACCAACTAAA

Aquaporin TIP2 FWD CAAGCCCACCTCCAATAAGT

Aquaporin TIP2 REV TGAACCCAGCTCGATCATTT

Zeaxanthin epoxidase FWD CTTTCTGGACAGTTCCGTAGAG

Zeaxanthin epoxidase REV AAAGCCATAGACCCACCTTG

Glutathione S-transferase FWD GGTATCACCACCAAAGTAGCA

Glutathione S-transferase REV CAACAAAGGAAGGCACAGAAAG

Nitrate reductase FWD TGGCAGCATGGTGGTATAAG

Nitrate reductase REV GCGTTGATGGGCAAGATTTC

Protein phosphatase FWD GAGCAACGACTTGACCAGAT

Protein phosphatase REV CTTGACGAACCTCCACTTCTT
