# Supplementary material for: Transcriptome assembly, profiling and differential gene expression analysis of the halophyte Suaeda fruticosa provides insights into salt tolerance
Source: BMC Genomics. 2015 May 6;16(1):353. doi: 10.1186/s12864-015-1553-x (PMC4422317; doi:10.1186/s12864-015-1553-x)
Supplement: Additional file 3: — Codes for bioinformatics analysis. [file 12864_2015_1553_MOESM3_ESM.pdf]

Supplementary file

Codes for Bioinformatics analysis

Quality trimming and Digital Normalization

Trimmomatic

```
java -jar trimmomatic -0.27.jar PE leftuntrimmed.fastq rightuntrimmed.fastq lefttrimmed.fastq s1_se.fastq righttrimmed.fastq s2_se.fastq ILLUMINACLIP: illuminaClipping.fa:2:30:10
```

Interleave paired end reads

```
python interleave-reads.py left.pe.fq right.pe.fq | gzip -9c > reads.pe.fq.gz
```

Fastq quality trimmer and Fastx trimmer

```
for i in *.pe.fq.gz *.se.fq.gz
do
   echo working with $i
   newfile="$(basename $i .fq.gz)"
   gunzip -c $i | fastq_quality_filter -Q33 -q 30 -p 50 | gzip -9c > "${newfile}.qc.fq.gz"
done
```

Digital Normalization

```
#Extracting paired ends from the interleaved files
for i in *.pe*.qc.fq.gz
do
   python strip-and-split-for-assembly.py $i
done

#Digital Normalization
python normalize-by-median.py -p -k 21 -C 30 -N 4 -x 3e9 --savehash normC30k21.kh *.pe.qc.fq.gz

#Trim erroneous k-mers
python filter-abund.py -V normC30k21.kh *.keep

#Strip and split orphaned and paired end- reads
for i in *.pe*.qc.fq.gz
do
   python strip-and-split-for-assembly.py $i
done
```

De Novo Assembly

Assembly with Velvet

```
velveth velvet.41 41 -fastq -short reads.se.qc.keep.abundfilt.fq.gz -shortPaired reads.pe.qc.keep.abundfilt.fq.gz \
velvetg velvet.41 -read_trkg yes -ins_length 300 -min_contig_lgth 200 -cov_cutoff 5 \
```

Scaffolding with Oases

```
oases velvet.41 -scaffolding yes -unused_reads yes -ins_length 300 -min_trans_lgth 200 -cov_cutoff 5 \

#performed for k-mers 35 to 99
```

Assembly with Trinity

```
ulimit -s unlimited
ulimit -a
Trinity.pl \
--seqType fq \
--JM 120G \
--output Trinityresults \
--SS_lib_type FR \
--CPU 20 \
--min_kmer_cov 2 \
--left left.fastq \
--right right.fastq \
--single single.fastq \

1> trinity.out \
2> trinity.err \
```

Make a SAM file (like those produced by GSNAP, bowtie2, SOAP2, etc) into a sorted, indexed BAM file for use by BamBam and other programs:

```
samtools view -Sb file.sam > file.bam
samtools sort file.bam file
samtools index file.bam
```

Call differential expression between 3 reps of 2 samples, using the EdgeR package (download from CRAN):

```
counter -g gene_annotation.gff sample1_rep1.bam sample1_rep2.bam sample1_rep3.bam sample2_rep1.bam sample2_rep2.bam sample2_rep3.bam > expression.txt
edgeR.R expression.txt differential_expression.txt
# modify the line "groups <- factor(c(1,1,1,2,2,2))" in edgeR.R to support other numbers os samples and reps
```

```
# Note: using the devel versions of both packages!
source("http://bioconductor.org/biocLite.R")
biocLite ("biomaRt")
biocLite ("edgeR")
biocLite ("DESeq")
library (edgeR)
setwd("~/Downloads")
library(DESeq) # version 1.9.11
library(edgeR) # version 2.99.8
library(VennDiagram)
library (biomaRt)

# Read in data ------------------------------------------------------------

## Use data
x <- read.delim("expressionoasescdhit45.txt", row.names=1, sep="\t", stringsAsFactors=FALSE)
head(x)
dim(x)

## Make metadata data.frame
meta <- data.frame(
  row.names=colnames(x),
  condition=c("0mM", "0mM", "0mM", "300mM", "300mM", "300mM", "0mM", "0mM", "0mM", "300mM", "300mM", "300mM"),
  libType=c("root", "root", "root", "root", "root", "root", "shoot", "shoot", "shoot", "shoot", "shoot", "shoot"))
meta$condition <- relevel(meta$condition, ref="0mM")
meta

## Independent filtering?
keep_cpm <- rowSums(cpm(x)>1) >=3
keep_quantile <- rowSums(x)>quantile(rowSums(x), probs=.5)
??probs
addmargins(table(keep_cpm, keep_quantile))
x <- x[keep_cpm, ]

# DESeq -------------------------------------------------------------------

## Make a new countDataSet
d <- newCountDataSet(x, meta)

## Estimate library size and dispersion
d <- estimateSizeFactors(d)
d <- estimateDispersions(d, method="blind", fitType="local")
?estimateDispersions
plotDispEsts(d, main="DESeq: Per-gene dispersion estimates")

## Principal components biplot on variance stabilized data, color-coded by condition-librarytype
print(plotPCA(varianceStabilizingTransformation(d), intgroup=c("condition", "libType")))

## Fit full and reduced models, get p-values
dfit1 <- fitNbinomGLMs(d, count~libType+condition)
dfit0 <- fitNbinomGLMs(d, count~libType)
```

```
dpval <- nbinomGLMTest(dfit1, dfit0)
dpadj <- p.adjust(dpval, method="BH")

## Make results table with pvalues and adjusted p-values
dtable <- transform(dfit1, pval=dpval, padj=dpadj)
dtable <- dtable[order(dtable$padj), ]
head(dtable)
dtable

# edgeR ------------------------------------------------------------------
## Make design matrix
condition <- relevel(factor(meta$condition), ref="0mM")
libType <- factor(meta$libType)
edesign <- model.matrix(~libType+condition)
edesign
## Make new DGEList, normalize by library size, and estimate dispersion allowing possible trend with average count size
e <- DGEList(counts=x)
e <- calcNormFactors(e)
e <- estimateGLMCommonDisp(e, edesign, verbose=TRUE)
e
e <- estimateGLMTrendedDisp(e, edesign)
e <- estimateGLMTagwiseDisp(e, edesign)
e

## MDS Plot
plotMDS(e, main="edgeR MDS Plot")

## Biological coefficient of variation plot
plotBCV(e, cex=0.4, main="edgeR: Biological coefficient of variation (BCV) vs abundance")
e$samples

## Fit the model, testing the coefficient for the 0mM and 300mM comparison
efit <- glmFit(e, edesign)
efit <- glmLRT(efit, coef="condition300mM")
FDR <-p.adjust (efit$table$PValue, method="BH")
sum (FDR <0.05)
## Make a table of results
etable <- topTags(efit, n=nrow(e))$table
etable <- etable[order(etable$FDR), ]
head(etable)
summary (etable)
top <- topTags(efit)
top
?cpm
??decideTestDGE
cpm(e)[rownames(top), ]
summary(de <- decideTestsDGE(efit))
head (de)
isDEr <-as.logical (de)
rDEnames <-rownames (efit$table[isDEr,])
rDEnames
Rtable <- data.frame (efit$table[isDEr,], FDR=p.adjust(efit$table$PValue, "BH") [isDEr])
Rtable <-Rtable [order(Rtable$FDR),]
rDEnames2 <- rownames (Rtable[Rtable$FDR <0.05 & abs(Rtable$logFC) > 2,])
rDEnames2
detags <-rownames(e)[as.logical(de)]
plotSmear (efit, de.tags=detags)
head(de)

topTags (efit, n=100)

## ~MA Plot
with(etable, plot(logCPM, logFC, pch=20, main="edgeR: Fold change vs abundance"))
with(subset(etable, FDR<0.05), points(logCPM, logFC, pch=20, col="red"))
abline(h=c(-1,1), col="blue")
```
